# Histone Modifications as an Intersection Between Diet and Longevity

**DOI:** 10.3389/fgene.2019.00192

**Published:** 2019-03-12

**Authors:** Diego Molina-Serrano, Dimitris Kyriakou, Antonis Kirmizis

**Affiliations:** ^1^UMR 6290, Centre National de la Recherche Scientifique, Rennes, France; ^2^Institute of Genetics and Development of Rennes (IGDR), Université de Rennes 1, Rennes, France; ^3^Efevre Tech Ltd., Larnaca, Cyprus; ^4^Department of Biological Sciences, University of Cyprus, Nicosia, Cyprus

**Keywords:** diet, histone modification, lifespan, aging, longevity, high-fat diet, low-protein diet, caloric restriction

## Abstract

Histone modifications are key epigenetic regulators that control chromatin structure and gene transcription, thereby impacting on various important cellular phenotypes. Over the past decade, a growing number of studies have indicated that changes in various histone modifications have a significant influence on the aging process. Furthermore, it has been revealed that the abundance and localization of histone modifications are responsive to various environmental stimuli, such as diet, which can also affect gene expression and lifespan. This supports the notion that histone modifications can serve as a main cellular platform for signal integration. Hence, in this review we focus on the role of histone modifications during aging, report the data indicating that diet affects histone modification levels and explore the idea that histone modifications may function as an intersection through which diet regulates lifespan. A greater understanding of the epigenetic mechanisms that link environmental signals to longevity may provide new strategies for therapeutic intervention in age-related diseases and for promoting healthy aging.

## Introduction

Biological aging or senescence is a multidimensional process that is manifested by degradation of biological function over time, which in the meantime confers susceptibility to different diseases (Cătană et al., 2018). A plethora of factors, both environmental and intrinsic, can affect aging. It is well accepted that a healthy diet and lifestyle exerts a positive effect on aging (Liang et al., 2018), while exposure to multiple stresses can have the opposite outcome (Amdam, 2011). Dietary choices during every stage of life, from embryo to old age, can significantly impact our growth and health (Upadhyaya et al., 2017). Many nutrients of food and derivatives produced during food digestion and metabolism have been well described for their roles in building and sustaining healthy organisms. On the contrary, severe food deprivation in developing countries or excess fat intake in the developed westernized world are known to negatively affect health (Nowacka-Woszuk et al., 2018; Uchiyama et al., 2018). Those diets lead to disorders such as cardiovascular diseases and diabetes, which dramatically affect people’s lifespan and well-being. The effect of diet on the human body was traditionally observed by changes in physiology at the organ level (e.g., fatty liver, atheromatic plaques, heart failures) or at the organismal level (obesity). However, for a long time it was unclear how diet affects the cells at the molecular level and, in turn, how these changes impact on lifespan.

The molecular mechanisms underlying the aging process have been the focus of various studies in the last few decades. Recently, nine tentative genetic and biochemical hallmarks of aging have been established: genomic instability, telomere attrition, altered intercellular communication, loss of proteostasis, deregulated nutrient sensing, mitochondrial dysfunction, cellular senescence, stem cell exhaustion, and epigenetic alterations (López-Otín et al., 2013). Among those, the epigenetic alteration hallmark has been attracting much interest over the last years for two main reasons: (1) there is increasing evidence linking epigenetic changes to age-related disorders, and (2) the potential reversibility of epigenetic marks makes them promising therapeutic targets to alleviate age-related pathologies (Milagro et al., 2013).

Cells respond to different nutrients through various signaling pathways, which eventually converge onto chromatin in order to regulate the expression of multiple genes involved in the specific cellular response. During aging, changes in gene expression have been consistently reported (de Magalhães et al., 2009; Hu et al., 2014; Rangaraju et al., 2015), and these changes can be deleterious or protective depending on the targeted gene and how its expression is regulated. However, although the signaling pathways that allow the cells to respond to specific environmental inputs are well-studied, substantial knowledge regarding the integration of the different signals at the chromatin level is still lacking. The basic unit of chromatin is the nucleosome, a dynamic structure consisting of 147 base pairs of DNA wrapped around an octamer of histone proteins. This octamer comprises two copies of each of the core histones: H2A, H2B, H3 and H4. Histones can be decorated with various post-translational modifications (PTMs), including acetylation, methylation, phosphorylation, and ubiquitination, and these PTMs are deposited and removed by specialized histone modifying enzymes (Bannister and Kouzarides, 2011). Due to this characteristic, epigenetic modifications are reversible and thus able to dynamically modulate chromatin structure in order to activate or silence gene expression. Furthermore, histones usually possess multiple PTMs, which can communicate among themselves to influence the presence of each other. These communications are known as cross-talks, and happen in a context-dependent manner, orchestrating a more complex transcriptional output than each PTM in isolation (Murr, 2010; Molina-Serrano et al., 2013).

**FIGURE 1:**
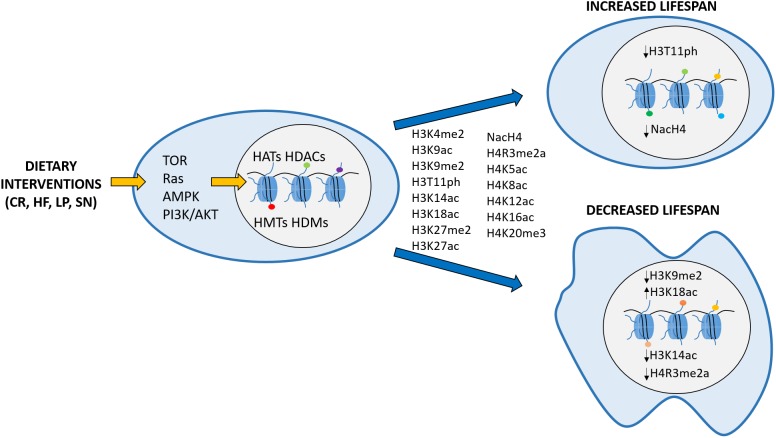
Schematic representation depicting the hypothesis that histone modifications act as an intermediate between diet and longevity. Calorie restriction (CR), high fat (HF), low protein (LP), single nutrient (SN) conditions are sensed by the cell through signaling pathways like TOR, Ras, AMPK or PI3K/AKT, promoting changes on the epigenome. Examples of histone modifications that are affected by dietary interventions are indicated between the two blue arrows. Specific changes in some of those modifications (H3K9me2, H3K18ac, NacH4, H3T11ph, H3K14ac, and H4R3me2a) have been consistently linked to a particular lifespan effect and are shown within the respective cell nucleus. Arrows next to each modification point to increase (↑) or decrease (↓) in the corresponding modification levels.

Several studies in various organisms demonstrated that environmental inputs could influence histone PTM deposition, thereby modulating chromatin structure and gene expression. For this reason, histone modifications are an excellent interface through which extracellular signals, like dietary manipulations or stress, may affect cellular phenotypes (Badeaux and Shi, 2013; Gut and Verdin, 2013). The effect of epigenetic changes on the aging phenotype has been studied extensively in the last decades. Hence, in this review, we summarize the knowledge acquired regarding the role of histone PTMs in regulating aging, and present the evidence linking dietary interventions to PTMs, some of which are then directly affecting lifespan (Figure 1).

## Histone Levels and PTMs are Linked to Lifespan Regulation

Histones and many of their PTMs undergo changes in their levels during aging, affecting different genomic regions in various tissues. This often complicates the interpretation on their specific effects on lifespan. There are three possible scenarios on how histones and their PTMs could affect lifespan: they can cause global changes in the transcriptome, alter the expression of key longevity genes or utilize both of these mechanisms (Cheung et al., 2010; Han et al., 2012; Sun et al., 2014; Ashapkin et al., 2017). Global histone loss during aging and senescence has been repeatedly described from yeast to humans (Feser et al., 2010; Tsurumi and Li, 2012; Hu et al., 2014). The implication of histone loss in aging was initially supported by the observation that in yeast, histone overexpression extends lifespan (Feser et al., 2010). Together with the drop of nucleosome occupancy (up to 50% in *Saccharomyces cerevisiae*) derived from the histone loss during aging, there is a marked redistribution of the remaining nucleosomes (Hu et al., 2014). This generates a general upregulation of many genes and an increase in genome instability that are both possible causes for cellular aging. Consistently, studies in *Drosophila melanogaster* have shown that decreased heterochromatin levels and thus reduction of nucleosomes are directly related to aging, whilst an increase in heterochromatin promotes longevity. This phenotype is associated with nucleosomal changes at the ribosomal DNA locus, thus affecting rRNA synthesis (Larson et al., 2012). Despite the important role of histone levels in aging, a large body of work in aging research has focused on histone PTMs and the enzymes that mediate them, as it was observed that manipulation of their levels can trigger substantial changes in lifespan. During the last decade, an increasing number of histone residues and PTMs have been linked to aging in various eukaryotic organisms (Table 1). However, this task has shown to be challenging due to the difficulty to isolate lifespan phenotypes from the plethora of cellular mechanisms affected by the experimental dietary interventions. For this reason, the baker’s yeast *S. cerevisiae* has served as an excellent model to test the role of histone residues in lifespan, as it allows for easy genetic manipulation, therefore obtaining causal links between histone point mutations and longevity. Following this concept, systematic, high-throughput lifespan studies in yeast using the H3/H4 histone mutant library showed that many histone residues, which can also bear modifications, could potentially affect lifespan, although still more in-depth studies are needed to better understand the aging effects of the associated PTMs (Sen et al., 2015). The recent establishment of a similar H2A/H2B histone mutant library could expand the number of histone residues that are associated with lifespan regulation, as these histones remain currently less studied (Jiang et al., 2017).

**Table 1:** Histone modifications and lifespan.

Histone
modification	Action	Lifespan	Organism	Citation
H3K4me	Deletion of several subunits of the H3K4 methyltransferase complex COMPASS	Decreased	*S. cerevisiae*	Smith et al., 2008; Ryu et al., 2014;Cruz et al., 2018
	Knockdown of several subunits of the H3K4 methyltransferase complex ASH-2	Increased	*C. elegans*	Greer et al., 2010
	Overexpression of the H3K4 demethylase RBR-2	Increased	*C. elegans*	Greer et al., 2010
	Mutation/knockdown of the H3K4me3 demethylase RBR-2	Decreased	*C. elegans*	Greer et al., 2010; Maures et al., 2011
	Mutation/Knockdown of the H3K4 demethylase RBR-2	Increased	*C. elegans*	Lee, 2003; Ni et al., 2012; Alvares et al., 2014
	Mutation of the H3K4 demethylase Lid	Decreased	*D. melanogaster*	Li et al., 2010
	Knockdown of the H3K4 demethylase LSD-1	Increased	*C. elegans*	McColl et al., 2008; Maures et al., 2011
H3K9ac	Overexpression of H3K9ac deacetylase SIRT6	Increased	*M. musculus*	Kanfi et al., 2012
	Deletion of H3K9ac deacetylase SIRT6	Decreased	*M. musculus*	Mostoslavsky et al., 2006; Kawahara et al., 2009
H3K9me	Deletion of the H3K9 methyltransferase SUV39H1	Decreased	*M. musculus*	Djeghloul et al., 2016
	Overexpression of the H3K9 methyltransferase SUV39H1	Decreased	*M. musculus*	Djeghloul et al., 2016
	Deletion of the H3K9 methyltransferase SUV39H1	Increased	*M. musculus* (Progeria)	Liu et al., 2013
H3T11ph	H3T11 mutant	Increased	*S. cerevisiae*	Oh et al., 2018
	Deletion of H3T11 Ser/Thr kinase CK2	Increased	*S. cerevisiae*	Oh et al., 2018
H3K14ac	H3K14 mutant	Decreased	*S. cerevisiae*	Xu et al., 2016
H3K18ac	Deletion of the H3K18 deacetylase SIRT6	Decreased	*H. sapiens*	Tasselli et al., 2016
H3K27me	Knockdown of the H3K27 demethylase UTX-1	Increased	*C. elegans*	Maures et al., 2011
	Mutation of the H3K27 methyltransferase PRC-2	Increased	*D. melanogaster*	Siebold et al., 2010
H3S28ph	H3S28 mutant	Increased	*D. melanogaster*	Joos et al., 2018
H3K36me	H3K36 mutant	Decreased	*S. cerevisiae*	Sen et al., 2015
	Deletion of H3K36 methyltransferase Set2	Decreased	*S. cerevisiae*	Sen et al., 2015
	Deletion/Inactivation of H3K36 methyltransferase MET-1	Decreased	*C. elegans*	Pu et al., 2015
	Deletion of H3K36 demethylase Rph1	Increased	*S. cerevisiae*	Sen et al., 2015
	Deletion of H3K36 demethylase JMJD-2	Increased	*C. elegans*	Ni et al., 2012
	Deletion of H3K36 methyltransferase SET-18	Increased	*C. elegans*	Su et al., 2018
H3K56ac	H3K56 mutant	Decreased	*S. cerevisiae*	Dang et al., 2009; Feser et al., 2010
	Deletion of H3K56 acetyltransferase Rtt109	Decreased	*S. cerevisiae*	Feser et al., 2010
	Deletion of H3K56 deacetylases Hst3 and Hst4	Decreased	*S. cerevisiae*	Feser et al., 2010
H3K79me	H3K79 mutant	Decreased	*S. cerevisiae*	Sen et al., 2015
	Deletion of H3K79 methyltransferase Dot 1	Decreased	*S. cerevisiae*	Ryu et al., 2014
NacH4	H4S1 mutant	Increased	*S. cerevisiae*	Molina-Serrano et al., 2016
	Deletion of the NacH4 acetyltransferase Nat4	Increased	*S. cerevisiae*	Molina-Serrano et al., 2016
H4R3me	H4R3 mutant	Decreased	*S. cerevisiae*	Molina-Serrano et al., 2016
H4K12ac	Mutation of the H4K12ac acetyltransferase Chameau	Increased	*D. melanogaster*	Peleg et al., 2016
H4K16ac	H4K16 mutant	Decreased	*S. cerevisiae*	Dang et al., 2009
	Deletion of the H4K16 deacetylase Sir2	Decreased	*S. cerevisiae*	Kaeberlein et al., 1999; Dang et al., 2009
	Deletion of the H4K16 acetyltransferase Sas2	Increased	*S. cerevisiae*	Dang et al., 2009
	Overexpression of the H4K16 deacetylase Sir2	Increased	*S. cerevisiae, C. elegans, D. melanogaster*	Kaeberlein et al., 1999; Tissenbaum and Guarente, 2001; Rogina and Helfand, 2004
	Deletion of the H4K16 deacetylase Rpd3	Increased	*S. cerevisiae, D. melanogaster*	Kim et al., 1999; Jiang et al., 2002;Rogina et al., 2002
H2BK123ub	H2BK123 mutant	Decreased	*S. cerevisiae*	Rhie et al., 2013
	Deletion of H2BK123 ubiquitinase complex Rad6/Bre1	Decreased	*S. cerevisiae*	Rhie et al., 2013
	Deletion of H2BK123 deubiquitinase Ubp10	Decreased	*S. cerevisiae*	Rhie et al., 2013
	Deletion of H2BK123ub SAGA/SLIK deubiquitinase subunits Sgf73, Ubp8 and Sgf11	Increased	*S. cerevisiae*	McCormick et al., 2014


### Histone H3 PTMs Related to Lifespan

#### H3K4

Lysine 4 on histone H3 can be found in one of three possible methylated forms: mono, di or trimethylated (H3K4me1, -2, and -3).The trimethylated form usually localizes at the promoter of actively transcribed genes (Santos-Rosa et al., 2002), and has been shown to have a strong implication with aging in several model organisms. In *S. cerevisiae*, deletion of different components or catalytic inactivation of the H3K4 methyltransferase complex COMPASS reduces significantly replicative lifespan (Smith et al., 2008; Ryu et al., 2014; Cruz et al., 2018). This effect is due to the impaired expression of several genes that are normally induced during aging. In *Caenorhabditis elegans* however, RNAi knockdown of several components of the H3K4me3 methyltransferase ASH-2 complex extend lifespan (Greer et al., 2010). This lifespan extension is dependent on the presence of H3K4me3 demethylase RBR-2, as deletion of RBR-2 abrogates the lifespan extension effect generated by the ASH-2 knockdown. Greer et al. (2010) also found that overexpression of RBR-2 extends lifespan, strongly suggesting that these two opposing chromatin modifiers must operate in the same lifespan-related pathway. However, results on the effect of RBR-2 are contradictory, as different reports have shown that mutation or RNAi-mediated knockdown of this enzyme both increases (Lee, 2003; Ni et al., 2012; Alvares et al., 2014) and decreases *C. elegans* lifespan (Greer et al., 2010; Maures et al., 2011). In *D. melanogaster*, RNAi depletion or mutation of Lid, the RBR-2 ortholog, causes an increase in the H3K4me3 levels and a reduction in male fly lifespan. Interestingly, under the same manipulation lifespan of female flies remains unperturbed (Li et al., 2010). Another H3K4 demethylase, LSD-1, has also been associated with lifespan. LSD-1 targets mono and dimethylated (H3K4me1 and H3K4me2, respectively) residues, and its depletion in *C. elegans* has been reported to extend lifespan compared to WT (McColl et al., 2008; Maures et al., 2011).

#### H3K9

Histone H3 lysine 9 can be acetylated (H3K9ac) and methylated (H3K9me), and both modifications have been connected to aging. While acetylation of this residue is usually found in active chromatin (Karmodiya et al., 2012), di and trimethylation (H3K9me2 and H3K9me3, respectively) usually mark constitutive heterochromatin and inactive euchromatin (Peters et al., 2001; Rougeulle et al., 2004). H3K9ac has been shown to increase during aging in *D. melanogaster* (Peleg et al., 2016), but decrease with age in rat liver cells (Kawakami et al., 2009). In mice this mark is deacetylated by SIRT6, a sirtuin that controls the expression of multiple glycolytic genes and triggers age-associated degenerative processes when is depleted (Mostoslavsky et al., 2006; Kawahara et al., 2009; Zhong et al., 2010). In line with this evidence, SIRT6 overexpression increased male longevity in transgenic mice, regulating specific genes in a similar way as when mice follow a CR diet (Kanfi et al., 2012). On the other hand, H3K9me3 and H3K9me2 have been shown to decrease during aging in fibroblast cells, *C. elegans* and *D. melanogaster* (Scaffidi and Misteli, 2006; Larson et al., 2012; Ni et al., 2012). Another report in *D. melanogaster* showed a general increase in H3K9me3 due to a more widespread distribution of this mark in the genome, including euchromatic regions (Wood et al., 2010). More recently, Djeghloul et al. (2016) have shown that the ability of hematopoietic stem cells to generate B lymphocytes is dependent on the H3K9 methyltransferase SUV39H1. The levels of this enzyme and H3K9me3 decrease with age in these cells, contributing to the aging-related decrease in immune function and this effect has been associated with changes in heterochromatin regulation. Accordingly, overexpressing SUV39H1 in aging cells improves the generation of B lymphocytes (Djeghloul et al., 2016).However, in progeroid mouse cells depletion of SUV39H1 reduces H3K9me3 levels and this delays aging due to restored DNA repair capacity. Remarkably, when SUV39H1 is deleted in combination with the metalloprotease ZMPSTE24, those mice have a 60% increase in lifespan (Liu et al., 2013).

#### H3T11

Histone H3 threonine 11 can be phosphorylated (H3T11ph) by different protein kinases and plays a role in several cellular functions, including meiosis (Govin et al., 2010), DNA replication checkpoint (Shimada et al., 2008), and nutritional stress (Oh et al., 2018). The levels of this modification are regulated by the Sch9 signaling pathway in response to glucose levels in food. H3T11 then regulates the expression of several genes implicated in the nutritional response. Interestingly, phosphorylation of this residue is implicated in *S. cerevisiae* aging, as mutating H3T11 to alanine clearly extends yeast chronological lifespan (Oh et al., 2018). Importantly, deletion of *CKA1*, the catalytic unit of the CK2 Serine/Threonine kinase complex that targets H3T11 shows the same effect. H3T11A and *cka1Δ* phenotypes seem to be caused ultimately by an increased tolerance to media acidification. In normal conditions, glucose depletion increases the levels of acetic acid, promoting phosphorylation of H3T11. This mechanism is obviously not functional in the mutants, changing the transcriptional output of the cell in a way that causes cellular longevity. The mechanism by which this histone phosphorylation affects transcription is, however, still elusive(Oh et al., 2018).

#### H3K14

In *S. cerevisiae*, H3K14 acetylation has been described to have a role in rDNA silencing and replication-dependent nucleosome assembly (Xu et al., 2016). Mutation of this residue markedly reduced yeast replicative lifespan, a phenotype enhanced when other residues in the histone H3 tail, such as K9, K18, and K23, that are acetylated are also mutated in combination with H3K14 (Xu et al., 2016).

#### H3K18

In mammals, acetylation at H3K18 (H3K18ac), a mark enriched at gene promoters and associated with active sites (Wang et al., 2008), is also involved in aging. H3K18ac is frequently found at pericentric chromatin, where is removed by SIRT6 (Kanfi et al., 2012). H3K18ac removal allows silencing of pericentric heterochromatin preventing mitotic errors and cellular aging. On the contrary, absence of SIRT6 increases H3K18ac thus, stimulating aberrant accumulation of pericentric transcripts, triggering acute aging (Tasselli et al., 2016).

#### H3K27

H3K27 can be found in one of three possible methylated forms: mono, di or trimethylated (H3K27me1, 2 and 3), and its trimethylated form is strongly associated with heterochromatin (Pan et al., 2018). In *C. elegans*, H3K27me3 levels have been observed to decrease with aging (Jin et al., 2011; Ni et al., 2012), correlating with the increasing levels of the H3K27me3 demethylase UTX-1 (Jin et al., 2011). Accordingly, knockdown of UTX-1 extends worm lifespan (Maures et al., 2011). A similar effect has been observed in mammalian cells, where UTX-1 levels increase with aging in parallel to a decrease in H3K27me3 (Jin et al., 2011). Other reports using human TIG3 cell cultures showed a decrease in H3K27me3 and its associated methyltransferase PRC2 complex in senescent cells(Bracken et al., 2007). In mouse brain H3K27me3 also decreases with age (Gong et al., 2015). However, another article also reported both upregulation and downregulation of H3K27me3 at the promoter of different genes in aged murine hematopoietic stem cells (Sun et al., 2014). In flies and killifish, however, H3K27me3 seems to affect lifespan differently from mammals and *C. elegans*. In killifish, PRC2 and H3K27me3 levels increase during aging (Baumgart et al., 2014), whereas heterozygous mutations in the catalytic subunit of the PRC2 complex reduced H3K27me3 levels and increased lifespan in *D. melanogaster* (Siebold et al., 2010). Consistent with this evidence, mutations in the Polycomb-antagonist complex TRX suppressed the lifespan extension of the PRC2 mutant while increasing H3K27me3 levels (Siebold et al., 2010). TRX is a complex that promotes H3K27 acetylation (H3K27ac), a modification that counteracts H3K27 methylation, further supporting the implication of this histone residue in the regulation of lifespan. Why H3K27 methylation states have a different effect on aging in the various organisms studied remains quite puzzling. This discrepancy could be a consequence of the regulation of distinct sets of longevity genes in the studied organisms, a difference in the conditions used during each of these studies or a combination of the two reasons as well as other yet unknown factors.

#### H3S28

Histone H3 serine 28 can be found in a phosphorylated form (H3S28ph), and this PTM cross-talks with H3K27 PTMs to respond to stress signaling by displacing the polycomb complex from its target genes (Gehani et al., 2010). Interestingly, Joos et al. (2018) have shown in a recent report that flies expressing ectopically a H3S28A mutant have increased longevity and improved stress resistance through the regulation of well-established longevity genes.

#### H3K36

H3K36 can also be found in three possible methylated forms but only its trimethylated state has been strongly associated with aging. H3K36me3 localizes at gene bodies, and it is associated with active transcription (Bannister et al., 2005). In *S. cerevisiae* H3K36me3 levels decrease during aging, triggering cryptic transcription (Sen et al., 2015); a pattern also observed in *D. melanogaster* and *C. elegans* (Wood et al., 2010; Pu et al., 2015). H3 lysine 36 substitutions performed in *S. cerevisiae* showed decreased lifespan and this effect was consistently observed in the absence of the H3K36 methyltransferase Set2 (Ryu et al., 2014; Sen et al., 2015). Interestingly, deletion of the H3K36 demethylase Rph1 had the opposite effect (Sen et al., 2015). Set2 is required for proper function of the TOR pathway and thus implicated in regulating the transcriptional response to nutrient stress (McDaniel et al., 2017). In agreement with this, depletion or inactivation of the Set2-ortholog MET-1 in *C. elegans* shortened lifespan (Pu et al., 2015), while depletion of the demethylase JMJD-2 enhanced longevity (Ni et al., 2012). More recently, deletion of the H3K36 dimethyltransferase SET-18 in *C. elegans* has been shown to extend lifespan through changes in the expression of DAF-16, a forkhead transcription factor that mediates the insulin/IGF conserved pathway(Su et al., 2018). Intriguingly, expression of SET-18 is upregulated in muscle cells of old worms, showing concomitant decrease in daf-16 expression.

#### H3K56

H3K56 is a residue involved in nucleosome assembly, genomic stability and transcription, and can be found both in acetylated and methylated forms (Das et al., 2009; Yuan et al., 2009; Jack et al., 2013). In *S. cerevisiae* H3K56 acetylation levels decrease with age, and mutating the residue greatly reduces lifespan (Dang et al., 2009; Feser et al., 2010; Sen et al., 2015). Furthermore, deletion of Rtt109, the main acetyltransferase targeting this residue, shortens yeast lifespan, an effect also observed when deleting the H3K56-associated deacetylases Hst3 and Hst4 (Feser et al., 2010). The fact that both loss and increase of this acetylation has the same effect on lifespan possibly reflects the implication of this residue and of its modification in various molecular mechanisms.

#### H3K79

H3K79, just like other lysine residues, can exist in one of three possible methylated forms: mono, di or trimethylated, which are involved in many cellular pathways, including transcription regulation, telomeric silencing, cell-cycle checkpoint, DNA repair, and cellular development (Farooq et al., 2016). While H3K79me3 has been shown to increase with age in yeast (Rhie et al., 2013), mutating the lysine residue into glutamic acid (H3K79E) has an evident reduction in replicative lifespan (Sen et al., 2015). Deletion of Dot1, the H3K79 methyltransferase also reduces lifespan (Ryu et al., 2014). In contrary to what has been observed in yeast, in aged mouse brain this modification is reduced (Gong et al., 2015).

### Histone H4 PTMs Associated With Lifespan

#### NacH4

N-terminal acetylation is present in all core histones, but is more abundant on histones H4 and H2A, catalyzed by the N-terminal acetyltransferase Nat4 (aka NatD, Naa40). This histone acetyltransferase has high substrate selectivity and its enzymatic activity is conserved from yeast to human (Ree et al., 2018). This modification crosstalks with the adjacent arginine 3 asymmetric dimethylation mark (H4R3me2a) to regulate ribosomal RNA expression in response to glucose levels suggesting that Nat4 and NacH4 act as a sensor for cell growth (Schiza et al., 2013). Mutating H4R3 into lysine (H4R3K) severely reduces yeast replicative lifespan, while loss of NacH4 by H4S1 mutation or Nat4 deletion extends yeast replicative lifespan in a similar manner to calorie restriction (CR). Constitutive expression of Nat4 counteracts the longevity effect induced by CR through a mechanism that involves the induction of specific stress-response genes and nicotinamidase Pnc1, a major regulator of Sir2 activity (Molina-Serrano et al., 2016).

#### H4K12

H4K12 can be found both in acetylated and methylated forms. In its acetylated form this residue is associated with active transcription (Wang et al., 2008). Interestingly, Peleg and colleagues found that old mice have deregulated H4K12ac levels in certain key genes, impairing their ability to learn (Peleg et al., 2010). Following this observation, they also show that recovering physiological levels of this modification by treatment with the histone deacetylase inhibitor SAHA restores the expression of learning-induced genes and the cognitive abilities of old mice. In another report, Peleg and colleagues also found that impairing Chameau, the main H4K12 acetyltransferase in *D. melanogaster*, promotes longevity and reduces age-related phenotypes(Peleg et al., 2016).

#### H4K16

H4K16 is usually found in an acetylated form and it is implicated in nucleosome–nucleosome interactions as well as higher order chromatin structure. This modification is present in active chromatin (Kimura et al., 2002; Suka et al., 2002) and its levels increase during aging (Dang et al., 2009). H4K16ac is removed by Sir2/SIRT1, a histone deacetylase with well-documented implications in aging (Houtkooper et al., 2012). Yeast mutant strains in which lysine 16 on H4 has been substituted by arginine (H4K16R) or glutamine (H4K16Q) show a decrease in replicative lifespan (Dang et al., 2009; Kozak et al., 2010). Consistently, deletion of the H4K16 deacetylase Sir2 has been extensively reported to shorten replicative lifespan in yeast (Kaeberlein et al., 1999; Dang et al., 2009), and deletion of Sas2, the major H4K16 acetyltransferase, has the opposite effect (Dang et al., 2009; Kozak et al., 2010). Along the same lines, evidence shows that Sir2 overexpression increases lifespan in yeast, worms and flies (Kaeberlein et al., 1999; Tissenbaum and Guarente, 2001; Rogina and Helfand, 2004), although this effect seems to be dependent on the genetic background (Kaeberlein et al., 2004; Burnett et al., 2011). In the brain of aged mice, SIRT1-associated genes are found to be deregulated, and overexpression of this enzyme can suppress this age-dependent deregulation (Oberdoerffer et al., 2008). Moreover, mice mildly overexpressing this enzyme have a better overall health, with lower levels of DNA damage and fewer spontaneous carcinomas (Herranz et al., 2010). Interestingly, deletion of Rpd3, another H4K16 deacetylase has the opposite effect to Sir2 deletion, increasing lifespan in yeast and *D. melanogaster* (Kim et al., 1999; Jiang et al., 2002; Rogina et al., 2002). However, while heterozygous flies have extended longevity, the complete removal of Rpd3 in *D. melanogaster* is lethal (Rogina et al., 2002). Whether the contradicting effects observed between Rpd3 and Sir2 are a consequence of targeting histones located on different genes is open to discussion. In mice, homozygotic mutants of the enzyme ZMPSTE24 exhibit reduced H4K16 acetylation and premature aging features. Notably, supplementing the drinking water of these mice with the histone deacetylase inhibitor sodium butyrate delays premature cellular aging in these mutants and increases lifespan (Krishnan et al., 2011).

### Histone H2B PTMs Associated With Lifespan

Histone H2B monoubiquitination at lysine 123 in yeast and, correspondingly, lysine 120 in vertebrates is required for the trimethylation of both H3K4 and H3K79 (Nakanishi et al., 2009). Interestingly, the levels of this H2B modification increase in replicative aged cells (Rhie et al., 2013). Yeast cells expressing a H2BK123R mutant are short-lived, a result reproduced by deleting the components of the H2B ubiquitinase complex Rad6/Bre1 and the deubiquitinase Ubp10 (Rhie et al., 2013). Moreover, strains carrying a deletion of some components of the SAGA/SLIK histone deubiquitinase complex, including Sgf73, Ubp8 and Sgf11 are long lived. This effect is specific to these three complex subunits, as deletion of other components of SAGA/SLIK have normal or shortened replicative lifespan (McCormick et al., 2014). Interestingly, the lifespan extension observed by deletion of each of these three components is dependent on Sir2 and linked to H4K16 acetylation as well as methylation at H3K4 and H3K79 residues (Rhie et al., 2013).

## Dietary Influences on Histone PTMs

Environmental factors such as exercise (Denham et al., 2014), circadian rhythms (Orozco-Solis and Sassone-Corsi, 2014) and even sexual stimuli (Promislow and Kaeberlein, 2014) are shown to influence gene expression and longevity in different organisms. However, nutrient availability and diet is to date the most thoroughly studied environmental factor to affect longevity. Additionally, diet is known to significantly affect the epigenome. Specifically, Wolff et al. (1998) showed for the first time that pregnant female mice fed with a methyl donor rich diet produced higher percentage of offspring with wild-type color coat compared to the ones that fed on a control diet. Since then, the scientific community working within this field was interested to determine whether diet can affect chromatin structure and gene transcription through epigenetic changes. In fact, histone PTMs are excellent intermediaries to connect changes between the environmental input (nutrient availability) and the biological output (transcriptional regulation) (Badeaux and Shi, 2013). Different dietary interventions like high-fat (HF) diet, low protein (LP) diet, and CR showed that extreme dietary conditions affect multiple nutrient sensing pathways and can cause global histone modification changes (Donohoe and Bultman, 2012; Ribarič, 2012) (Table 2). However, even though dietary interventions, such as CR, have been shown to impact on lifespan, the causal evidence that establishes histone modifications as the molecular bridge between diet and longevity is still underdeveloped. Nevertheless, below we briefly introduce nutrient-signaling pathways that feed into histone modifications and then summarize the evidence which demonstrate that dietary interventions influence the occurrence of lifespan-associated histone modifications described above.

**Table 2:** Dietary influences on histone modifications and their cellular effects.

Dietary intervention	Affected locus	Effect on histone modification	Cellular effect	Organism	Citation
Caloric restriction	Stress-response genes	↓NacH4 ↑H4R3me2a	Increased *PNC1* expression	*S. cerevisiae*	Molina-Serrano et al., 2016
Caloric restriction	Stress-response genes	↑H3T11ph	Changes in oxidative stress response	*S. cerevisiae*	Oh et al., 2018
Caloric restriction	*Thrsp*	↓H3K9ac ↓H3K14ac ↓H4K5ac ↓H4K8ac ↓H4K12ac ↓H4K16ac	Decreased *Thrsp* expression	*Rattus norvegicus*	Shimada et al., 2011
Caloric restriction	*Glut4*	↑H4ac	Increased *Glut4* expression in adipose tissue	*M. musculus*	Wheatley et al., 2011
		↓H3K14ac ↑H3K9me2	Decreased *Glut4* expression in muscle (offspring)	*R. norvegicus*	Jiménez-Chillarón et al., 2012
Caloric restriction	*Igf1*	↓H3K4me2 ↑H3K4me3	Increased *Igf1* expression (offspring)	*R. norvegicus*	Jiménez-Chillarón et al., 2012
Caloric restriction	*p16(INK4A)*	↓H3ac ↓H3K4me2 ↑H3K9me3	Decreased expression of *p16(INK4A)* gene and Evasion of cellular aging (lung fibroblasts)	*Homo sapiens*	Li et al., 2011
Caloric restriction	*TERT*	↑H3ac ↑H4ac ↑H3K4me2	Increased *TERT* expression (Fetal lung fibroblasts)	*H. sapiens*	Daniel and Tollefsbol, 2015
High fat diet	*p16(Ink4a)*	↑H4ac ↓H3K27me	Increased *p16(Ink4a)* expression and hepatic cellular aging (obese rats)	*R. norvegicus*	Zhang et al., 2012
	*p21(Cip1)*	↑H3ac ↑H4ac ↑H3K4me2 ↓H3K27me2	Increased *p21(Cip1)* expression and hepatic cellular aging (obese rats)		
High fat diet	Global	↑H3K27ac	Increased gene expression of multiple genes	*M. musculus*	Siersbæk et al., 2017
		↓H3K27ac	Decreased gene expression of multiple genes		
High fat diet	Global	↓H3K23mal ↓H3K122ac ↓H3K18me2 ↓H3R26me1 ↓H2BK43me1 ↓H3R49me1 ↓H2AK118me1 ↓H3K18bu ↓H2BK108me2 ↓H3K23pr ↑H4K31me1 ↑H4R35me1 ↑H3R128me1 ↑H3K36me2 ↑H3R36me1	Extensive hepatic gluconeogenesis (prediabetic mice)	*M. musculus*	Nie et al., 2017
High fat diet during gestation and lactation	*p16(Ink4a)*	↓H4ac	Decreased *p16(Ink4a)* expression in the mammary gland (offspring)	*R. norvegicus*	Zheng et al., 2012a
High fat diet during gestation	*Pck1*	↓H3ac ↓H3K4me2 ↓H3K9me3	Increased gluconeogenic genes expression and high blood glucose levels (offspring)	*R. norvegicus*	Strakovsky et al., 2011
High fat diet during gestation	*Adipoq*	↑H3K9me	Decreased adiponectin levels in adipose tissue (offspring)	*M. musculus*	Masuyama and Hiramatsu, 2012
	Lep	↑H4K20me	Increased leptin levels in adipose tissue (offspring)		
High fat diet during gestation	*TRβ*	↑H3K9ac ↑H3K14ac	Increased *TRβ* expression in fetal liver cells (offspring)	*Macaca fuscata*	Suter et al., 2012
High fat diet during gestation	*Pon1/Pon3*	↑H4ac ↑H3K4me2	Increased *Pon1 and Pon3* expression in liver (male offspring)	*M. musculus*	Strakovsky et al., 2014
Low protein diet during gestation	*Igf2*	↓H3K4me3 ↓H4K20me3 ↑H3K9me3 ↑H3K27me3	Decreased *Igf2* expression in liver (fetus)	*M. musculus*	Strakovsky et al., 2011
Low protein diet during gestation	*Cyp7a1*	↓H3K9ac ↑H3K9me3	Decreased *Cyp7a1* expression and hyper-cholesterolemia from post-natal to adulthood (offspring)	*R. norvegicus*	Sohi et al., 2011
Low protein diet during gestation	*p21(Cip1)*	↓H3 acetylation ↓H3K4me2	Decreased *p21(Cip1)* expression in mammary gland	*R. norvegicus*	Zheng et al., 2012b
Low protein diet during gestation	*G6PC*	↓H3K9me3 ↑H3 acetylation ↑H3K4me3	Increased *G6PC* expression in liver (offspring, Gender specific effect)	*Sus scrofa*	Jia et al., 2012
Low protein diet during gestation and lactation	*MSTN*	↑H3K9ac ↑H3K4me3	Increased *MSTN* expression (offspring)	*S. scrofa*	Jia et al., 2016
Low protein diet during embryo preimplantation	*Gata6*	↓H3ac ↓H4ac	Decreased *Gata6* expression (embryonic stem cells)	*M. musculus*	Sun et al., 2015
Curcumin	Global	↑H4ac	Decreased *HDAC1, HDAC3* and *HDAC8* expression in Raji cells (Burkitt’s lymphoma)	*H. sapiens*	Liu et al., 2005
	Global	↓H3ac ↓H4ac	Glioblastoma cancer cells and adult neural-derived stem cells		Park et al., 2012
PEITC	Global	↑H3ac ↑H3K4me ↓H3K9me3	Decreased HDAC activity and expression (Human prostate cancer cells)	*H. sapiens*	Wang et al., 2007
EGCG	*GSTP1*	↑H3K9ac ↑H3K18ac ↑H4ac	Decreased HDAC activity and restoration of GSTP1 expression (Human prostate cancer cells)	*H. sapiens*	Pandey et al., 2010
	*p21(CIP1) and* *p16(INK4A)*	↑H3K9ac ↑H3K14ac ↑H4K5ac ↑H4K12ac ↑H4K16ac ↓H3K9me	Increased expression of *p16(INK4A)* and *p21(CIP1)* (Sking cancer cells)		Nandakumar et al., 2011
Genistein	*p21(CIP1) and* *p16(INK4A)*	↑H3ac ↑H4ac	Increased *p21(CIP1)* and *p16(INK4A)* expression (Human prostate cancer cells)	*H. sapiens*	Majid et al., 2008


### Nutrient-Sensing Signaling Pathways Involving Histone Modifications

Sirtuins are probably the best-studied family of enzymes implicated in changing the epigenome as a response to environmental signals. Sirtuins are histone deacetylases (HDACs) that mediate lysine deacetylation through NAD hydrolysis, yielding *O*-acetyl-ADP-ribose nicotinamide. As NAD-dependent deacetylases, sirtuins are a perfect candidate to mediate lifespan response to nutrients, having a dual role as NAD-sensors and transcriptional regulators trough the deposition of PTMs in histones and other target proteins (Houtkooper et al., 2012). Sir2/SIRT1 is the most studied sirtuin in aging, and it has been extensively reported that raising its activity by altering NAD+/NADH levels, genetic manipulation or chemical stimulation robustly extends lifespan in yeast, *C. elegans*, *D. melanogaster* and mammals (Guarente, 2013). This effect is not exclusive of Sir2/SIRT1, as other members of this family have also been consistently implicated in lifespan regulation (Grabowska et al., 2017). NAD+ levels increase during fasting or exercising, while HF diet reduces the NAD+/NADH ratio (Cantó et al., 2010; Kim et al., 2011). The NAD+ levels would therefore influence the deacetylation of H3K9, H3K56 and H4K16, among other sirtuin targets with strong effect on lifespan regulation.

Target of Rapamycin (TOR) is a well-preserved nutrient sensing pathway that has been extensively linked to CR and lifespan regulation, and its inhibition through rapamycin increases lifespan in several organisms (Johnson et al., 2013). Many yeast proteins (and their orthologs in higher eukaryotes) acting downstream the Tor pathway, like Sch9 and Rim15 kinases, the stress-response transcription factors Msn2/4 and the H3K36 HDMs Gis1 and Rph1 have been linked to lifespan regulation (Pedruzzi et al., 2000; Fabrizio et al., 2001; Kaeberlein et al., 2005; Wei et al., 2008; Sen et al., 2015). The H3K36 methyltransferase, Set2, is also required for TOR signaling and regulates the transcriptional response to nutrient stress (McDaniel et al., 2017). TOR shares many downstream components with the glucose-sensing Ras/PKA, another pathway linked to lifespan regulation. The histone deacetylase Sir2 is phosphorylated by this pathway, which then regulates its ability to mediate lifespan under CR conditions (Kang et al., 2015). In mammals, the cAMP/CREB signaling pathway, which is activated during short term fasting (Altarejos and Montminy, 2011), relies on the PKA kinase to promote CREB association with CBP/p300, a coactivator family with the ability to acetylate histones (Bannister and Kouzarides, 1996; Ogryzko et al., 1996). Interestingly, the cAMP/CREB pathway has been the focus of studies regarding the cognitive deficits that appear with aging (Hansen and Zhang, 2013).

AMPK/Snf1 is another energy sensing pathway induced by ATP depletion that regulates transcription through many epigenetic functions. The AMPK/Snf1 kinase complexes can be translocated from the cytoplasm into the nuclei upon activation, where they can regulate the activity of several transcription factors (Salminen et al., 2016). AMPK/Snf1 is a histone kinase itself that phosphorylates yeast H3S10 and mammalian H2BS36 residues (Lo et al., 2005; Bungard et al., 2010). AMPK/Snf1 also regulates the activity of several histone acetyltransferases and deacetylases through phosphorylation of these enzymes (Salminen et al., 2016). This pathway also affects histone acetylation and deacetylation by controlling acetyl CoA and NAD+ levels.

The insulin signaling pathway, conserved from *C. elegans* to mammals, has been extensively associated with aging in different organisms. Mutations of its different components regulate genes that are involved in lifespan (Kenyon, 2011), and genetic interventions that decrease growth hormone and insulin-like growth factor signaling robustly extend longevity in mammals (Brown-Borg, 2015). The PI3K/AKT pathway is central to insulin signaling and can phosphorylate several chromatin-modifying enzymes such as the histone acetyltransferase p300, the H3K27 methyltransferase EZH2 and BMI1, a core component of the polycomb repressive complex 1 associated with H2AK119 ubiquitination (Cha et al., 2005; Huang and Chen, 2005; Nacerddine et al., 2012).

### Effects of Dietary Interventions on Lifespan-Associated Histone Modifications

#### Caloric Restriction

Among the various dietary interventions, CR is the best characterized, and it can increase lifespan in yeast, worm, flies, rodents, and primates. In primates, CR can delay the onset of long-lasting age-related diseases such as cardiovascular disease, type 2 diabetes, degenerative diseases and cancer (Ribarič, 2012). CR is shown to extend lifespan even under alternate day fasting (ADF), where animals were fed with normal diet 1 day and caloric restricted diet the next day (Ribarič, 2012). The beneficial effects of CR arise from multiple mechanisms, including reduction of oxidative stress, modulation of metabolic pathways and, interestingly, chromatin remodeling (Jiménez-Chillarón et al., 2012). Here we provide an extensive overview of the links to histone modifications (Table 2).

In yeast, CR has been shown to regulate the expression of stress response genes through changes in the modification of key histone residues. N-terminal acetylation on histone H4 (NacH4) and the phosphorylation of H3T11 are two histone residues that have been linked to CR-mediated pathways. Both PTMs regulate lifespan through the transcriptional regulation of different stress-response genes. Specifically, CR decreases the acetylation levels at the N-terminus of histone H4. This, in turn, upregulates several stress-response genes, including the one encoding for nicotinamidase Pnc1, a main regulator of Sir2 activity and a well-characterized gene involved in replicative lifespan (Molina-Serrano et al., 2016). CR also affects H3T11 phosphorylation, in this case enhancing the modification. This mechanism regulates the expression of several stress-response genes, which eventually affect chronological lifespan by influencing cell tolerance to media acidification (Oh et al., 2018). These are two of the best examples of histone modifications through which a dietary input (CR) affects the expression of key regulators that eventually impact on cellular longevity.

CR stimulates several epigenetic effects in mammals. Rats fed with a diet containing an indigestible starch showed a reduction of the key transcription factor ChREBP (carbohydrate response element binding protein) and THRSP (thyroid hormone-responsive spot 14 protein) levels. The promoter/enhancer and transcribed regions of the *Thrsp* gene fed with indigestible starch showed a decrease in acetylation in the H3K9, H3K14, H4K5, H4K8, H4K12, and H4K16 residues compared with rats fed with digestible starch (Shimada et al., 2011). Another effect of CR is the increase in histone H4 acetylation on the *Glut4* (glucose transporter 4) gene promoter in adipose tissue of obese mice. GLUT4 is responsible for regulating glucose metabolism through uptake into adipocytes, and the increased acetylation enhances its expression. Moreover, 50% CR during gestation in rats reduced GLUT4 in offspring muscles. These reduction is concomitant with a decrease in H3K14ac and an increase in H3K9me2 (Jiménez-Chillarón et al., 2012). A 50% CR diet in rats during gestation also showed epigenetic changes in the offspring liver. Those changes included an increase in H3K4me3 and a decrease in the H3K4me2 levels at the Igf1 locus, a gene essential for the insulin signaling pathway (Jiménez-Chillarón et al., 2012). In human cell cultures glucose restriction reduces the expression of the *p16(INK4A)* gene. This is accompanied by a decrease in H3ac and H3K4me2 and an increase in H3K9me3 promoter levels (Li et al., 2011). Additionally, glucose restriction increased H3ac, H4ac and H3K4me2 levels on the *Tert* promoter and increased its expression in human fetal lung fibroblasts (Daniel and Tollefsbol, 2015). Free-feeding (*ad libitum*) long-lived rats have the same gene expression profile as the ones fed with CR. The overlapping expression profile contained genes that encode for proteins involved in histone acetylation and deacetylation such as NUR77 (NR4A1) and mortality-like factor 1 (MORF4L1), as well as other additional chromatin regulators like BAG4, MEF2C, HDAC5, BCOR11, H3F3B, BRD3, KAT6A, HIST1HD AND CHMP1A. These proteins may be implicated in the dietary memory effect (Wood et al., 2015).

CR seems to work through several pathways, including sirtuin activation (Herranz and Serrano, 2010; Li et al., 2011), inhibition of lipogenesis, regulation of the mitochondrial function and glucose homeostasis. SIRT1 acts as a metabolic sensor of CR and can delay aging by providing increased stress resistance through regulation of the tumor-suppressor protein p53 and the fork head box O gene FOXO3a. SIRT1 deacetylates histones as well as non-histone proteins, such as the transcription factor NF-kB. Interestingly, many CR mimetic phytochemicals work as sirtuin activators (vel Szic et al., 2015). For example, resveratrol (RSV), a compound obtained from the skins of red grapes, is a caloric restriction mimetic which activates Sir2/SIRT1 in a dose dependent manner (Price et al., 2012). RSV increases lifespan in yeast, worms, flies, and fish (Price et al., 2012). In mammalian cells, RSV also activates the AMPK pathway and directly phosphorylates PGC-1α, the key regulator of energy metabolism. In human peritoneal mesothelial cells, RSV delays replicative aging by mobilization of antioxidant and DNA repair mechanisms (Ribarič, 2012). Other compounds like 2-deoxyglucose, a glucose analog that blocks glucose metabolism, can also induce SIRT1 in mammals (Davis and Ross, 2007).

#### High-Fat Diet (HF)

High-fat diet is another well characterized dietary intervention. Mice fed in these conditions may develop obesity, together with impaired glucose tolerance and enhanced pyruvate tolerance. Rats fed with HF diet that become obese (obesity prone – OP)show hepatic cellular aging. However, some mice develop resistance (obesity resistant – OR) and thus do not show the OP phenotype (Zhang et al., 2012). Interestingly, the liver cells of OP mice have increased *p16(Ink4a*) and *p21(Cip1)* mRNA levels compared to OR mice. These two genes are regulators of cellular aging, and their upregulation is associated with changes in PTM levels. The *p16(Ink4a*) gene showed higher H4ac and lower H3K27me levels at the promoter and/or the coding region in the liver of OP compared to OR mice (Zhang et al., 2012). Similar results have been observed in *p16(Ink4a*) expression when comparing OP to control mice (Zhang et al., 2018). This is one of the earliest examples of how diet affects histone modifications, which in turn impact on specific genes that regulate cellular lifespan (Zhang et al., 2012). However, the *p21(Cip1)* gene results are contradictory. While the *p21(Cip1)* gene showed an increase in H3ac, H4ac and H3K4me2 and decreased H3K27me3 levels at the promoter and/or the coding region in OP mice compared to OR mice (Zhang et al., 2012), the same author in a later report found that *p21(Cip1)* is decreased in HF-treated mice compared to control, with no significant PTM changes (Zhang et al., 2018). In recent reports, HF feeding in mice has been associated with reduced H3K18ac and H3K23ac levels in white adipose tissue and in the pancreas (Carrer et al., 2017), while hepatic gene transcription regulation is associated with changes in H3K27ac levels (Siersbæk et al., 2017). Interestingly, the effects of HF diet can be reversed in mice, as H3K27ac and global hepatic gene transcription levels return to similar levels to control after weight loss (Siersbæk et al., 2017). In another study using diet-induce obese mice (DIO) as prediabetic model, the authors analyzed the liver of these mice and found 10 significantly downregulated and 5 significantly upregulated PTMs compared to control (Nie et al., 2017). Particularly, H3K36me2 was increased 80% in DIO mice, suggesting a possible involvement in prediabetes development. Interestingly, H3K36me2 increase was reversed by metformin, a widely used drug for treating Type 2 Diabetes (Nie et al., 2017). HF diet has also been shown to affect considerably the expression of neuropeptides in the brain (hypothalamus and brain stem), which are responsible for controlling feeding behavior, energy metabolism and body-weight homeostasis (Milagro et al., 2013). Particularly, hypothalamic arcuate nucleus (ARH) neurons, which control feeding behavior in adult mice, show increased levels of histone deacetylases HDAC5 and HDAC8 when the mice are fed with long-term (4 weeks) HF diet (Funato et al., 2011).

HF diet effects are transmitted from embryonic stages into adulthood, regardless of the dietary choices later in life (Howie et al., 2009; Milagro et al., 2013). Maternal HF diet during gestation and lactation is responsible for a plethora of epigenetic changes in the offspring, and some of the HF diet hereditary epigenetic changes can last for up to two generations (Dunn and Bale, 2009). For example, timed-pregnant rats fed with a HF diet throughout gestation and lactation showed reduced *p16(Ink4a)* levels in the mammary gland. These changes were associated with reduced H4ac levels and increased HDAC3 recruitment within the *p16(Ink4a)* promoter region (Zheng et al., 2012a). The offspring of HF fed mother rats also have altered PTMs in their livers compared to the offspring from mothers fed with the control diet. Additionally, *Pck1*, the main control point for the regulation of gluconeogenesis, showed decreased H3ac, H3K4me2, H3K9me3 and H3K27me3 levels in HF conditions (Strakovsky et al., 2011). Adiponectin (regulator of glucose and fatty acid metabolism) expression in the adipose tissue was also reduced in the offspring of mice fed with HF diet during pregnancy, due to a decrease in H3K9ac and concomitant increase in H3K9me levels at the gene promoter (Masuyama and Hiramatsu, 2012). In the same report, the authors observed an increase in leptin levels (hormone, regulator of energy balance and inhibitor of hunger) in the adipose tissue, combined with increased H4K20me at the promoter of its gene (Masuyama and Hiramatsu, 2012). Other studies using pregnant young adult female Japanese macaques fed with a HF diet during pregnancy showed enrichment of the transcriptional coactivators MED1 (mediator complex subunit 1) and NCOA1 (nuclear receptor coactivator 1) on the *THRβ* (Thyroid hormone receptor B) gene in fetal liver. The *THRβ* gene promoter was also enriched by histone H3K9ac and H3K14ac, and the expression of THRβ was elevated compared to the offspring from mothers with control diet. Deregulation of thyroid hormones in the offspring of mothers fed with HF diet can lead to obesity (Suter et al., 2012). Interestingly, maternal HF diet can affect the offspring in a different manner based on their genders. Specifically, HF diet during pregnancy in mice gave birth to male offspring with high triglycerides and increased the expression of the antioxidant genes *Pon1* and *Pon3* compare to controls. On the contrary, in female offspring the changes in *Pon1* and *Pon3* mRNA levels were not statistically significant. The hepatic *Pon1* promoter had significantly more histone H4 acetylation and H3K4me2 in both genders. However, H3K9me3 was only associated with antioxidant genes in females (Strakovsky et al., 2014). Paternal HF diet changed the epigenome in spermatozoa and offspring liver. Specifically, under HF diet, H3K4me was enriched in paternal sperm around the transcriptional start site (TSS) of genes involved in development regulatory processes such as *Hoxd11, Hoxd13, Bai3, Foxp2*, and *Foxa2*. In contrast, in the offspring liver, H3K4me was enriched in genes controlling lipid biosynthesis, fatty acid synthesis and the oxidation-reduction process (Terashima et al., 2015).

#### Low-Protein (LP) Diet

Maternal malnutrition may lead to several epigenetic alterations in the offspring. For example, gestational protein restriction in mice decreased H3K4me3 and H4K20me3 while it increased H3K9me3 and H3K27me3 levels on the *Igf2* (Insulin-like growth factor-II) locus, resulting in its repression in fetal liver (Strakovsky et al., 2011). IGF2 deficiency in humans and mice leads to hepatic lipid homeostasis and growth restriction of the embryo (Strakovsky et al., 2011). LP diet in pregnant rats also led to hyper-cholesterolemia in postnatal rat offspring (d21), which persisted into adulthood (d130). Specifically, LP diet reduced the acetylation and increased the trimethylation of H3K9 at the *Cyp7a1* (Cholesterol 7 alpha-hydroxylase) gene promoter, causing its transcriptional repression in the rat offspring. The increase in H3K9me3 deposition was preceded by reduction of its demethylase Jmjd2 during fetal life. In embryonic stage (D19), H3K9me3 levels were not increased and *Cyp7a1* expression was modestly reduced (Sohi et al., 2011). Maternal diet during pregnancy can affect certain metabolic processes during the offspring’s mammary gland development and may predispose the offspring to breast cancer later in life. Accordingly, decrease of *p21(Cip1)* mRNA and protein levels was observed in the offspring mammary gland of Sprague–Dawley rats fed with LP diet during gestation. *p21(Cip1)* down-regulation was associated with reduced acetylation of H3 and H3K4me2 in its promoter (Zheng et al., 2012b).

Female pregnant pigs fed with LP diet gave birth to male piglets with hepatic activation of *G6PC* gene. These observations were combined with reduced H3K9me3 and increased histone H3 acetylation and H3K4me3 levels on the *G6PC* promoter compared with the offspring males from control diet fed mothers. Increase of *G6PC* gene expression in male piglets could possibly contribute to adult-onset hyperglycemia (Jia et al., 2012). LP diet in pigs during pregnancy and lactation caused upregulation of myostatin (MSTN) mRNA and protein levels in the muscle of male piglets 28 weeks of age. The MSTN increased levels were associated with enrichment of H3K9ac and H3K4me3 marks and increase of FOXO3 (forkhead box class O family member protein 3) and GR (glucocorticoid receptor) levels on the gene promoter of piglets from LP fed sows compared to the piglets from sows fed standard protein (Jia et al., 2016).

Maternal LP diet during preimplantation also affected epigenetically the embryos. Particularly, mice fed with LP diet prior to embryonic implantation caused reduction of expression of GATA6 transcription factor in the primitive endoderm of their embryos. The GATA6 expression change was linked to reduction of H3 and H4 acetylation and reduced RNA polymerase II levels on its promoter. Consistently, they also observed upregulation of the *Hdac1* gene (Sun et al., 2015).

#### Effects of Specific Nutrient Components

Several natural compounds from widely accessible food products can act as regulators of the epigenome. Sulforaphane, an isothiocyanate from broccoli and cabbage, and allylcompounds from garlic (diallyl disulfide and *S*-allyl mercaptocysteine) can have HDAC inhibitory effect. Phenethyl isothiocyanate (PEITC), present in cruciferous vegetables; and epigallocatechin-3-gallate (EGCG) from green tea are found to have HDAC inhibitory activity too. Another HDAC inhibitor, sodium butyrate, is found in cheese and butter and can regulate histone acetylation levels at the *p21(Cip1)* promoter (Davie, 2003). Also luteolin, found in parsley, thyme, peppermint, basil herb, celery and artichoke; and curcumin from turmeric can block HDAC activity. Additionally, curcumin has HAT inhibitory activity, since it binds the HAT p300 and causes conformational change in the enzyme, decreasing its affinity to histones H3 and H4 and acetyl CoA (Stefanska et al., 2012). Diindolylmethane, a digestion byproduct of indole-3-carbinol generated in the stomach from different vegetables (including broccoli, cabbage, cauliflower, mustard and radish) causes proteasomal degradation of the histone deacetylases HDAC1, HDAC2 and HDAC3. Chromatin immunoprecipitation (ChIP) experiments showed a reduction in the localization of these enzymes at the *p21(Cip1)* and *p27(Kip1)* gene promoters (Shankar et al., 2013). Dietary polyphenols such as RSV, quercetin and catechins have also an effect activating the HDAC Sir2/SIRT1. RSV binds to SIRT1 to cause a conformational change that allows tighter fluorophore holding and stronger peptide substrate binding (Borra et al., 2005). RSV also activates the HAT p300, which participates in the formation of an active chromatin structure (Narayanan et al., 2003). Despite the clear correlation between specific nutrients and activity or localization of particular histone modifiers, it is still not clear which substrates are affected in all these cases. More studies are required to determine whether molecular mechanisms are directed through histone modifications or through other non-histone substrates of the nutrient-regulated HDACs and HATs.

The epigenetic effects of particular nutrients are extensively studied in cancer cells. Curcumin downregulates p300 expression in B-cell non-Hodgkin lymphoma cell (Chen et al., 2007) and promotes the proteasome-dependent degradation of p300 and CBP (Shankar et al., 2013). Curcumin also inhibits the expression of HDAC1 and 2 in breast cancer or cervical cancer lines (Cătană et al., 2018), and of HDAC1, 3 and 8 in Raji cells while increasing H4 acetylation (Liu et al., 2005). Curcumin also decreases acetylation levels of H3 and H4 in glioblastoma cancer cells and adult neural-derived stem cells (Park et al., 2012). PEITC has been found to increase global levels of acetylated histone H3 and H3K4 methylation while decreasing the level of H3K9me3 in prostate cancer cells (Wang et al., 2007). EGCG decreases HDAC activity and blocks HDAC1-3 expression while raising H3K9ac, H3K18ac and H4ac levels in prostate cancer cells (Pandey et al., 2010). Other reports have shown that EGCG increases the expression of *p16(INK4A) p21(CIP1)* in skin cancer cells by downregulation of HDAC activity and increasing the H3K9ac, H3K14ac, H4K5ac, H4K12ac, and H4K16ac levels (Nandakumar et al., 2011). Diallyl disulfide inhibitory effects on HDACs are correlated to tumor suppression through *p21(CIP1)* induction in human colon cancer cells (Myzak and Dashwood, 2006; Nian et al., 2009; Stefanska et al., 2012). Sulforaphane mediates histone acetylation changes locally on *p21(Cip1)* and *Bax* gene promoters in mice and human colon and prostate cancer cells, thus being considered an important chemopreventive factor (Myzak et al., 2006; Dashwood and Ho, 2007). Particularly, single dose of sulforaphane can significantly inhibit HDAC activity and increase histone H3 and H4 acetylation levels globally and locally on those genes (Myzak et al., 2006). In addition, sulforaphane was also shown to inhibit HDAC activity in peripheral blood mononuclear cells (PBMCs) in humans (Myzak et al., 2007). The HDAC inhibitory action of luteolin is associated with the inhibition of cancer cell growth, survival and invasion in human epithelioid lung cancer cells (Attoub et al., 2011). Genistein, found in soy products, was found to increase H3ac, H4ac and H3K4me2 at the *p21(CIP1)* and *p16(INK4A)* transcription start sites, causing their reactivation in human prostate cancer cell lines (Majid et al., 2008). Finally hydroxycitrate (HCA), found in tropical plants, is a competitive inhibitor of the ATP citrate lyase. This enzyme has a role in histone acetylation and is involved in tumorigenesis and cancer cell metabolism through activation of the Akt pathway (Cătană et al., 2018).

## Concluding Remarks

Accumulating data demonstrate that diet affects the epigenome in a significant degree. Moreover, it is well established that histone modifications can regulate lifespan. However, only a handful of scientific reports showed a direct mechanistic link between diet and longevity through changes in histone PTMs. This demonstrates the difficulty in finding causal evidence that link dietary interventions to histone modification-associated mechanisms which subsequently alter lifespan, especially in mammals. One of the reasons for this obstacle is the lack of experimental models that allow for direct interrogation of the effects of histone mutants on the lifespan phenotype. It is not surprising that *S. cerevisiae*, with its advantage in genetic amenability and well-established protocols has helped the most in studying the direct role of PTMs in aging. Interestingly, various dietary interventions seem to affect modifications on histone residues that have already been linked to lifespan regulation in yeast such as H3K4me, H3K9ac, H3K9me, H3K14ac, H3K27me, and H4K16ac among others. This is perhaps a good indicator that histone PTMs serve as a crossroad between diet and longevity.

To date, CR is the best-described dietary intervention affecting longevity, mainly through changes in global histone acetylation levels and the regulation of multiple genes. Systematic, high-throughput studies showed that many histone residues could potentially regulate lifespan (Sen et al., 2015). Together with the fact that H2A and H2B histone modifications are substantially less studied, it is easy to hypothesize that other unidentified histone PTMs mediating the dietary effects on cell lifespan remain to be discovered. The detection of novel changes in histone modifications altered under different dietary interventions could be achieved through systematic mass spectrometry studies.

Intriguingly, one of the key features of epigenetic marks is their dynamic nature, opening the possibility to reprogram the epigenome with the aim to enhance our lifespan. Some conceivable ways to accomplish this could involve interventions in our diet and daily habits. Diet can regulate aspects such as lipid intake and NAD+ levels, promoting a lipid-specific gene expression profile (McDonnell et al., 2016), affecting histone acetylation by regulating sirtuin activity, and possibly changing the expression of longevity genes (Cantó et al., 2010). Another possibility would entail natural compounds with an effect on lifespan that could be supplemented at the right dose in our diet. For example, while high fat diet shows cellular aging in the liver of rodents (Zhang et al., 2012), RSV intake can correct this effect and extend the lifespan of these mice through AMPK/SIRT1 activation and improved insulin sensitivity (Donohoe and Bultman, 2012). The circadian clock, which has strong correlation with epigenetic mechanisms and nutrient sensing, could also contribute to enhance health span and healthy aging with simple changes in the sleep/wake cycles (Orozco-Solis and Sassone-Corsi, 2014). Another approach to lengthen our lifespan and improve our health would involve altering pharmacologically the epigenome of individuals to delay the aging process. Drugs like metformin and rapamycin can mimic some of the CR effects and showed very promising lifespan extension results in mice (Harrison et al., 2009; Martin-Montalvo et al., 2013). Accordingly, multiple therapeutic approaches have been explored with the aim to reprogram histone marks in order to avoid the appearance or progression of aging. Perhaps the most explored route is the usage of HDAC inhibitors to treat diseases like neurodegeneration, cancer, metabolic disorders and others (Tang et al., 2013). HMT inhibitors are also promising, and had shown beneficial effects in the memory of aging mice by targeting H3K9me3 (Snigdha et al., 2016). The results obtained with these drugs are quite promising, and thus many other inhibitors that target different histone modifying enzymes are nowadays under development (Helin and Dhanak, 2013).

The long-term goal of many studies is to decipher the right balance between exercise and personalized nutrition leading to beneficial epigenetic changes that will increase our health span. An advantage of epigenetic regulation, in addition to being reversible, is based on the fact that it can be inherited by the progeny helping to perpetuate the acquired longevity effects during several generations (Greer et al., 2011). However, this is a double-edged sword, as the opposite scenario is also possible, where the epigenetic consequences of choosing a bad diet could also be passed to our descendants (Huypens et al., 2016). The epigenetic component of the aging process is undeniably complex, but future studies could unravel the precise underlying mechanisms that could illuminate preventive or therapeutic dietary interventions in order to promotehealth and longevity.

## Author Contributions

All authors wrote the manuscript. DK and DM-S contributed equally to this work.

## Conflict of Interest Statement

DK was employed by company Efevre Tech Ltd. The remaining authors declare that the research was conducted in the absence of any commercial or financial relationships that could be construed as a potential conflict of interest.
